# Using cross-sector data linkage to track patient journeys across health and social care.

**DOI:** 10.23889/ijpds.v7i3.1785

**Published:** 2022-08-25

**Authors:** Fiona Grimm, Dan Lewer, John Craig, Rafi Rogans-Watson, Jenny Shand

**Affiliations:** 1 The Health Foundation; 2 UCL Collaborative Centre for Inclusion Health; 3 Care City Innovation; 4 University Hospitals Sussex; 5 UCLPartners

## Objectives

Older people and people with complex needs often require both health and social care services, but there is limited insight into individual journeys across these services. To help inform joint health and social care planning, we aimed to assess the relationship between hospital admissions and domiciliary care receipt.

## Approach

We used an individually linked dataset of primary care activity, hospital admissions and local authority-held social care records for adults living in Barking and Dagenham, a borough in London, England, on 1 April 2018, and followed them up until 31 March 2020. The outcome was initiation of a new domiciliary care package. We estimated the rate of hospital-associated care package initiation, and of care packages unrelated to hospital admissions. We also described the characteristics of hospital admissions that preceded domiciliary care and examined which primary diagnoses codes were associated with receiving domiciliary care after discharge.

## Results

In our cohort, 1.4 of participants had a domiciliary care package during a median follow-up of 1.87 years. One in three domiciliary care packages were initiated during a hospital stay or within 7 days of discharge. The rate of new domiciliary care packages was 120 times greater (95% CI 110-130) during or after a hospital stay than at other times, and this association was present for all age groups. Primary admission reasons accounting for the largest number of domiciliary care packages were hip fracture, pneumonia, urinary tract infection, septicaemia, and exacerbations of long-term conditions (COPD and heart failure). Admission reasons with the greatest likelihood of a subsequent domiciliary care package were fractures and strokes.

## Conclusion

Hospitals are a major referral route into domiciliary care. While new and acute illnesses account for many domiciliary care packages, exacerbations of long-term conditions and age- and frailty-related conditions are also an important driver. National-level linked datasets are needed for a better understanding of the relationship between health and social care receipt.

